# Evaluating bone quality and asymmetrical aplasia of the thoracic vertebral body in Lenke 1A adolescent idiopathic scoliosis using hounsfield units

**DOI:** 10.3389/fsurg.2022.1028873

**Published:** 2022-10-31

**Authors:** Taiqiu Chen, Wenjun Hu, Yan Peng, Yong Li, Jincheng Qiu, Xianjian Qiu, Pengfei Li, Shaoguang Li, Anjing Liang, Wenjie Gao, Dongsheng Huang

**Affiliations:** ^1^ Department of Orthopedics, Sun Yat-sen Memorial Hospital of Sun Yat-sen University, Guangzhou, China; ^2^ Department of Orthopedics, People’s Hospital of Jieyang, Jieyang, China; ^3^ Department of Radiology, Sun Yat-sen Memorial Hospital of Sun Yat-sen University, Guangzhou, China

**Keywords:** adolescent idiopathic scoliosis, bone quality, Hounsfield units, bone mineral density, spine

## Abstract

**Study Design:**

Retrospective analysis.

**Objective:**

To evaluate bone quality and investigate asymmetrical development of the thoracic vertebral body in adolescent idiopathic scoliosis (AIS) based on Hounsfield unit (HU) measurements obtained from computed-tomography (CT) scans.

**Summary of Background Data:**

HU value demonstrated higher reliability and accuracy than the traditional method, indicating that they could be used to individually evaluate and effectively assess the bone quality of every vertebra in the CT films.

**Methods:**

Total 30 AIS patients classified as Lenke Type 1A and 30 paired controls were included in this study. Regions of interest for HU value were measured on three horizontal images of the thoracic vertebrae. HU measurements of the whole vertebral body in each vertebra were obtained. Using HU value, we separately measured the concave and convex sides of each vertebral body in patients' group, as well as within the left and right sides in controls.

**Results:**

In controls, the mean HU value of T1–T12 thoracic vertebral bodies was 240.03 ± 39.77, with no statistical differences among different levels. As for AIS patients, in the structural curve, the apical region had a significantly lower HU compared with the other regions, and asymmetrical change was found between the concave and convex sides, most significantly in the apical region. In the non-structural curve, the average HU value was 254.99 ± 44.48, and no significant difference was found either among the different levels of vertebrae or between the concave and convex sides.

**Conclusions:**

Abnormal and asymmetrical changes in bone quality of the thoracic vertebral body in patients with Lenke 1A AIS were indicated. Low bone quality in the convex side of the structural curve indicated stronger internal fixation in surgery to correct the deformity.

## Background

Adolescent idiopathic scoliosis (AIS) is a complex three-dimensional deformity of the spine, characterized by lateral spinal curvature with a Cobb angle exceeding 10 degrees (1–4). The incidence of AIS is currently about 2%–3%, making it the most common spinal deformity in children (5). When untreated, progressive AIS is associated with restrictive lung disease, pain, severe deformity, and even mental health problems, posing a serious burden to the family and society (6, 7).

The causes of AIS are complex, including genetics, abnormal nervous-system function, endocrine abnormalities, biomechanical changes, and abnormal vertebral development (8, 9). Low bone quality had been found in AIS patients compared with healthy controls (10–17). AIS patients were reported to have poorer bone mineral density in bilateral femoral neck and central skeleton compared with controls (13, 14). Asymmetrical development of the vertebrae was also considered to be an important factor in the pathogenesis of AIS. Previous studies had established that longitudinal growth of the vertebral body in AIS patients was disproportionate (1, 9, 18). Asymmetrical changes in the width of thoracic pedicle in AIS patients vs. controls had also been found (19). However, only a few studies have evaluated the bone quality of the vertebral body in AIS patients.

The Hounsfield unit (HU) is a dimensionless unit generated from computed-tomography(CT) scans, which is obtained by linear transformation of the measured attenuation coefficient. HU value is considered an effective benchmark of bone quality (20–22). Compared with traditional methods, HU value permits more effective evaluation of the bone quality of every vertebral body, but it does not register the abdominal calcification that dual-energy x-ray absorptiometry scans cannot distinguish from attenuation (23–26). The purpose of our study is to evaluate bone quality and investigate asymmetrical development of the thoracic vertebral bodies based on HU measurements obtained from CT scans.

## Material and methods

### Subjects

Inclusion criteria for AIS patients were as follows: (1) careful screening to ensure that their scoliosis was idiopathic and classified as Lenke 1A (27); and (2) preoperative radiographs and CT images were available on file. Exclusion criteria for AIS patients were as follows: (1) proven or even suspected congenital, muscular, neurological, or hormonal cause of scoliosis; (2) receipt of spinal surgery or brace treatment; and (3) spinal infection or metabolic disease that could affect the accuracy of HU measurement. Inclusion criteria for controls were as follows: (1) gender, age, weight and height matched with patients; (2) clinical indications for CT (such as pneumonia) but no abnormal skeletal system findings assessed by a radiologist; and (3) no spinal bone infection or metabolic disease. Ultimately, 30 Lenke 1A AIS patients and 30 paired controls were included in our study. Therefore, total 30 structural curves (main thoracic curves) and 30 non-structural curves (proximal thoracic curves) were measured. Their demographic data were shown in Table 1.

**Table 1:** Demographic data of AIS patients and controls.

Demographic	Patients	Control subjects	*P*-value
Number	30	30	–
Gender	Female	Female	–
Age (years)	17.6 ± 3.40	17.8 ± 3.50	0.82
Height (cm)	156.3 ± 4.60	157.9 ± 2.90	0.13
Weight (kg)	44.8 ± 4.60	46.5 ± 3.40	0.08
BMI (kg/m^2^)	18.3 ± 1.40	18.4 ± 1.40	0.71
Cobb angle (°)	56.70 ± 20.20	–	–

### Data collection and assessment

**Figure 1:**
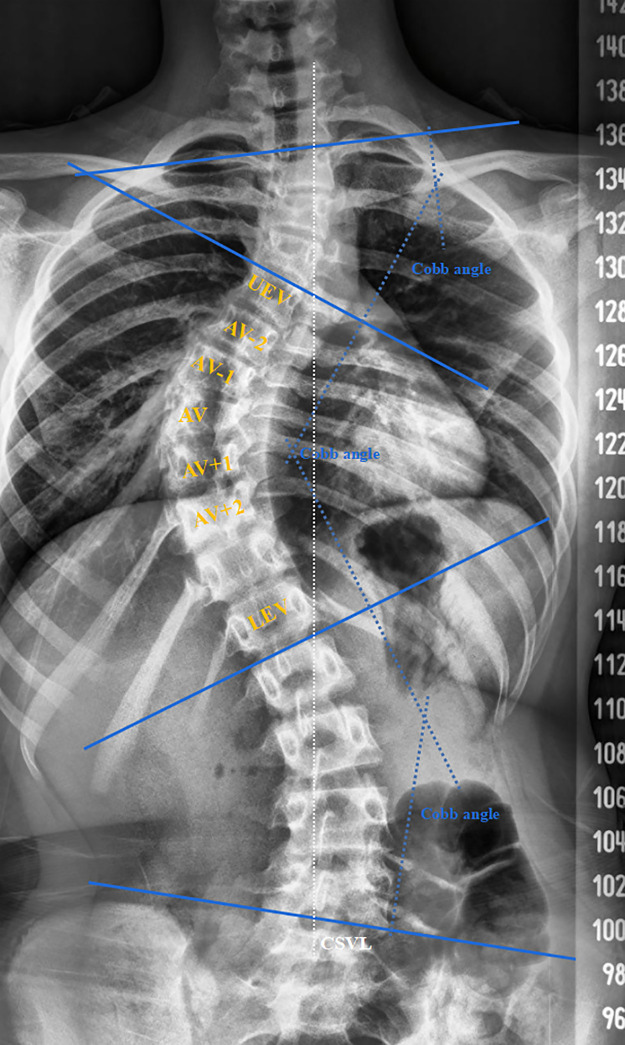
Measurement diagram of the AP x-ray of the whole spine. Three Cobb angles, including the structural and non-structural curves, was shown. The AV was defined as the vertebral body farthest from the CSVL. If the intervertebral disc was located at the farthest position, we collected data from the upper and lower vertebrae at the same time, bringing two apical vertebrae into one apical region. AV-1 was defined as the upper vertebra adjacent to AV; AV-2 was defined as the upper vertebra adjacent to AV-1; AV + 1 was defined as the lower vertebra adjacent to AV; AV + 2 was defined as the lower vertebra adjacent to AV + 1. The UEV and LEV were defined as the vertebrae with the largest inclinations at the head and at the tail of the curve respectively.

Demographic data, including age (year), height (cm), weight (kg) and body mass index (BMI; kg/m^2^) were collected. Standard whole-spine x-ray in the anteroposterior (AP), lateral and bending-position views were used. As shown in Figure 1, measurement of radiographic data mainly relied on the patient's whole-spine AP x-ray. We measured the Cobb angle and differentiated structural from non-structural curves by Lenke classification (27). A total 30 structural curves (main thoracic curves) and 30 non-structural curves (proximal thoracic curves) were measured. The apex vertebra (AV) was defined as the vertebral body farthest from the center sacral vertical line (CSVL). If the intervertebral disc was located at the farthest position, we collected data from the upper and lower vertebrae at the same time, bringing two apical vertebrae into one apical region. AV-1 was defined as the upper vertebra adjacent to AV; AV-2 was defined as the upper vertebra adjacent to AV-1; AV + 1 was defined as the lower vertebra adjacent to AV; AV + 2 was defined as the lower vertebra adjacent to AV + 1. The upper-end and lower-end vertebrae (UEV, LEV) were defined as the vertebrae with the largest inclinations at the head and at the tail of the curve respectively.

**Figure 2:**
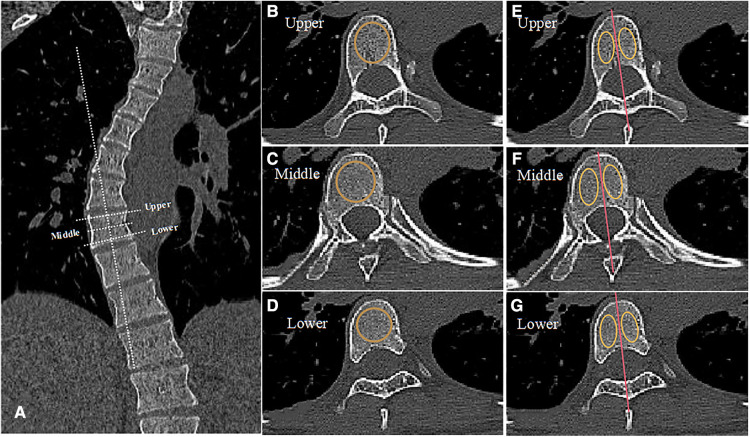
Measurement diagram of HU value. (**A**) The dashed white line represents the appropriate angulation on a reformatted workstation for obtaining the transverse CT image for each vertebra, displaying different planes. (**B–D**) The dotted orange circle represents the area we focused on in three different planes of the same vertebra: below the upper endplate of the vertebra, in the middle of the vertebra, and above the lower endplate of the vertebra. (**E–G**) We drew the red line to divide the vertebra into concave and convex sides through the spinous process as shown. The solid yellow circle represents the area we focused on for HU value measurement.

CT scans were performed on a 64-slice scanner (Toshiba Aquilion1 64-slice; Toshiba Medical Systems Corporation, Otawara-shi, Japan) at 120 kV and less than 200 mA, with a slice thickness of 0.5 mm and a resultant average radiation burden less than 10 mGy to reduce radiation exposure. During the scans, protections of sensitive glands were performed. Before taking measurements, 3D reconstruction of the CT film was performed and three suitable slices were obtained, as shown in Figure 2. The dashed white line represented the appropriate angulation on a reformatted workstation for obtaining the transverse CT image for each vertebra. HU value of the whole vertebral bodies, the concave and convex sides were separately measured at three locations of the vertebra on three horizontal planes: below the upper endplate of the vertebra, in the middle of the vertebra, and above the lower endplate of the vertebra. The solid yellow circle represented the areas that we focused on, which were used for HU measurement. The HU value of each vertebra was defined as the average HU value for all three planes. For each measurement, we drew the largest possible elliptical region of interest, excluding the cortical margins to prevent volume averaging.

### Statistical analysis

We analyzed all data using GraphPad Prism version 8.0.1 (GraphPad Software, San Diego, CA, USA) and SPSS version 20.0 (IBM Corp., Armonk, NY, USA). HU value among different vertebrae and degrees of variation in different regions were compared *via* one-way analysis of variance (ANOVA) followed by Bonferroni's *post hoc* test. We compared HU value between the concave and convex sides of each vertebra using the paired *t* test. The results were considered to be significant when two-way *P* < 0.05, and the range of agreement was defined as mean ± standard deviations (SDs).

## Results

### Vertebral-body bone quality in the apical region of the structural curve was decreased in AIS patients

**Figure 3:**
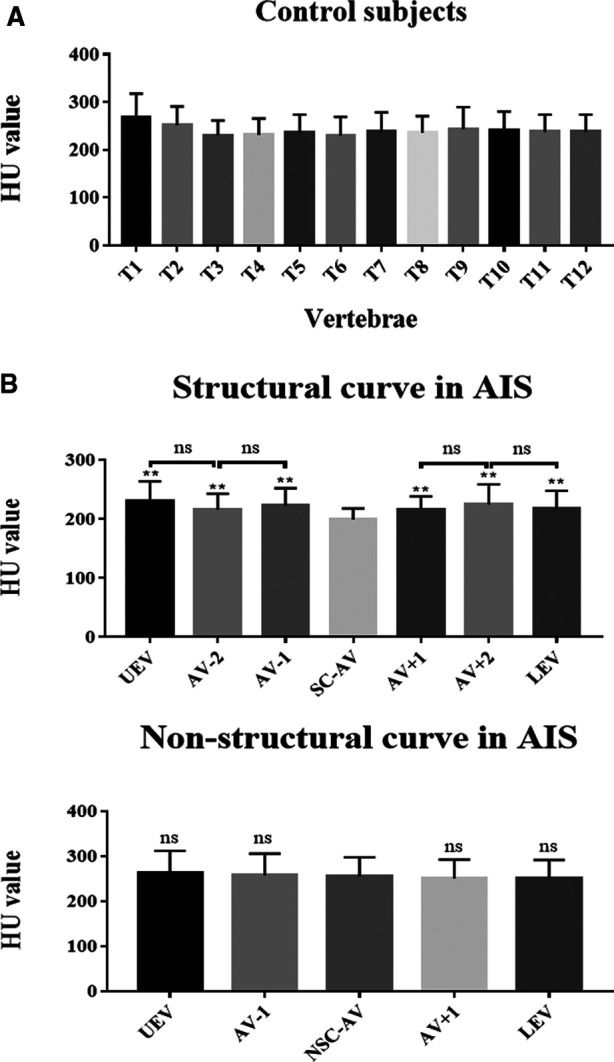
Vertebral-body bone quality of the apical region of the structural curve was decreased in AIS patients. (**A**) HU value of thoracic vertebral bodies from T1 to T12 in controls. (**B**) HU value of different levels of vertebral bodies in AIS patients, including the structural and non-structural curves. Total 30 patients with Lenke type 1A AIS and 30 paired controls were included in this study. ns: no statistical significance; **P < 0.01 vs. SC-AV or NSC-AV group.

A total of 30 patients with Lenke 1A AIS and 30 paired controls were included in our study. The HU value of T1-T12 thoracic vertebral bodies in controls were shown in Table 2. There was no significant difference among the different levels (Figure 3A). As for AIS patients, the HU value in the apical region of the structural curve was significantly lower than that in other regions (Table 3 and Figure 3B), but in the non-structural curve we found no significant difference among HU value in different regions (Table 3 and Figure 3B). Besides, we found that the average HU value of structural curve in Lenke 1A AIS patients was lower when compared to controls (Supplementary Figure S1). Meanwhile, we compared the average HU value between the structural and non-structural curves in AIS patients, and found that there was a statistically significant decrease in the regions of structural curves (Supplementary Figure S2).

**Table 2:** Hu value in controls.

Level	HU value
T-1	268.38 ± 49.56
T-2	251.26 ± 39.42
T-3	230.34 ± 30.90
T-4	231.22 ± 34.25
T-5	236.75 ± 36.98
T-6	229.96 ± 38.66
T-7	237.41 ± 41.41
T-8	235.27 ± 35.56
T-9	243.38 ± 46.11
T-10	241.31 ± 39.01
T-11	237.59 ± 36.24
T-12	237.50 ± 36.36
Average	240.03 ± 39.77

**Table 3:** Hu value in AIS patients. .

Structural curve		Non-structural curve	
Level	HU value	Level	HU value
UEV	229.60 ± 34.28**	UEV	261.41 ± 50.36
AV-2	215.75 ± 26.99**	AV-1	256.73 ± 48.92
AV-1	223.23 ± 29.03**	AV	254.85 ± 42.35
AV	199.40 ± 18.26	AV + 1	250.57 ± 41.93
AV + 1	216.38 ± 22.11**	LEV	251.42 ± 40.05
AV + 2	224.86 ± 33.92**	Average	254.99 ± 44.48
LEV	217.75 ± 30.09**		

UEV means upper-end vertebra; AV-2 means upper vertebra adjacent to AV-1; AV-1 means upper vertebra adjacent to AV; AV means apex vertebra; AV + 1 means lower vertebra adjacent to AV; AV + 2 means lower vertebra adjacent to AV + 1; LEV means lower-end vertebra. **P < 0.01 vs. AV group.

### Asymmetrical changes in vertebral-body bone quality in AIS patients

**Figure 4:**
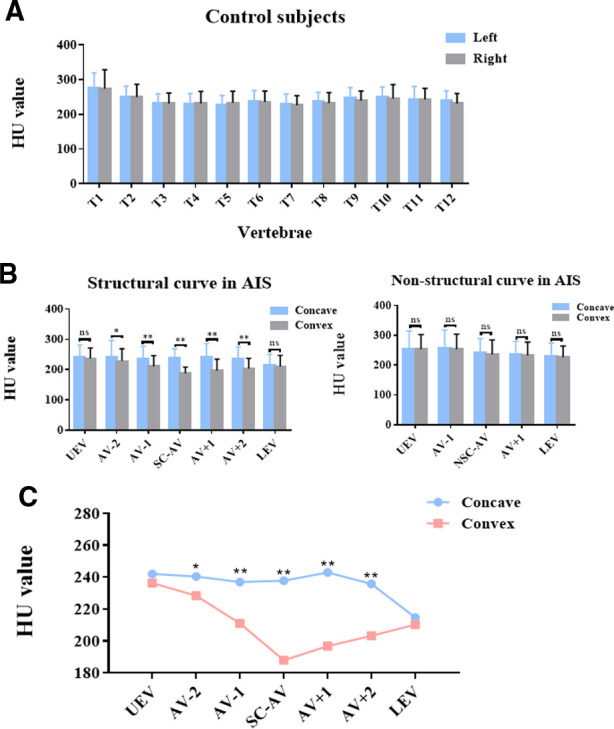
Asymmetrical changes in vertebral-body bone quality in AIS patients. (**A**) HU value on the left and right sides of thoracic vertebral bodies in controls. (**B**) HU value on the concave and convex sides of thoracic vertebral bodies in different regions of the structural and non-structural curves in AIS patients. (**C**) Comparison of HU values within the concave and convex sides of thoracic vertebral bodies in different regions of the structural curve in AIS patients. Total 30 patients with Lenke type 1A AIS and 30 paired controls were included in this study. ns: no statistical significance; *P < 0.05 vs. convex group, **P < 0.01 vs. convex group.

HU values were measured within the left and right sides of thoracic vertebral bodies in controls and within the concave and convex sides of thoracic vertebral bodies in AIS patients. As shown in Figure 4A, no significant difference in HU value was found between the left and right sides in controls (Figure 4A). As for AIS patients, the structural curve showed significant asymmetrical changes in HU values between the concave and convex sides in the AV-2, AV-1, AV, AV + 1, and AV + 2 regions but not in the UEV or LEV region. In the non-structural curve, no significant difference was found between the concave and convex sides in the UEV, AV-1, AV, AV + 1, or LEV region (Figure 4B). Besides, HU values in convex were lower than that in concave in AIS patient, and this difference could be more obvious in the apical region (Figure 4C).

### In AIS patients, asymmetrical changes in vertebral-body bone quality were most significant in the apical region

To compare the degree of asymmetrical change between the concave and convex sides in different regions of AIS patients, we calculated the variation degree of bone quality (VDBQ) as follows:

$$\begin{array}{rl} & \mathrm{VDBQ}\;(\text{\%})=\Sigma [\left(\mathrm{HU}\;\mathrm{value}\;\mathrm{of}\;\mathrm{concave}\;\mathrm{side}-\mathrm{convex}\;\mathrm{side}\right)\\ & \;/\;\mathrm{convex}\;\mathrm{side}]\mathrm{number}\;\mathrm{of}\;\mathrm{vertebrae}\;\mathrm{involved}\;\mathrm{in}\;\mathrm{the}\;\mathrm{region} \end{array}$$

**Figure 5:**
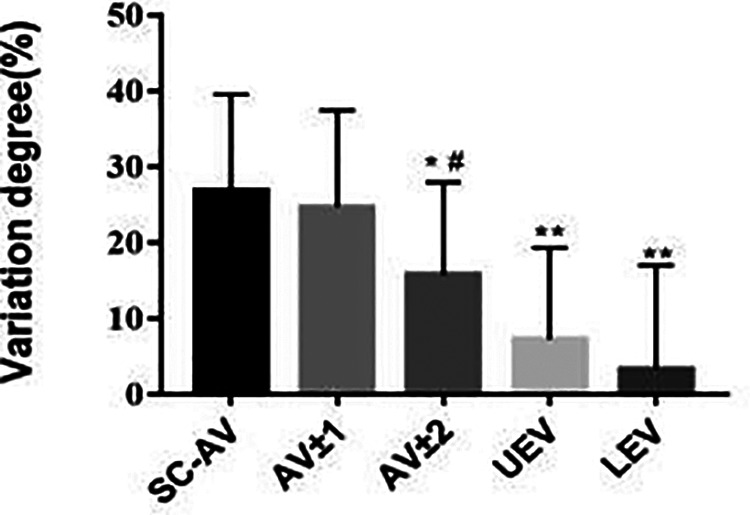
In AIS patients, asymmetrical changes in vertebral-body bone quality were most significant in the apical region. Comparison of variation degree of bone quality in different regions of the structural curve in AIS patients. Total 30 patients with Lenke type 1A AIS and 30 paired controls were included in this study. *P < 0.05 vs. SC-AV group, **P < 0.01 vs. AV group, ^#^*P* < 0.05 vs*.* AV ± 1 group.

As shown in Table 4 and Figure 5, we found that the VDBQ (%) in AV (26.82 ± 12.73) was higher than that in AV ± 2 (15.71 ± 12.24), UEV (7.28 ± 12.06) and LEV (3.30 ± 13.70), but we found no significant difference between AV (26.82 ± 12.73) and AV ± 1 (24.69 ± 12.73). The VDBQ in AV ± 1 (24.69 ± 12.73) was higher than that in AV ± 2 (15.71 ± 12.24), but no statistical difference among AV ± 2 (15.71 ± 12.24), UEV (7.28 ± 12.06) and LEV (3.30 ± 13.70) was found.

**Table 4:** Variation degree of bone quality in different regions of the structural curve in AIS patients.

Region	Variation degree (%)
AV	26.82 ± 12.73
AV ± 1	24.69 ± 12.73
AV ± 2	15.71 ± 12.24*,***
UEV	7.28 ± 12.06**
LEV	3.30 ± 13.70**

AV, apex vertebra; AV + 1, vertebra adjacent to AV; AV + 2, vertebra adjacent to AV + 1; UEV, upper-end vertebra; LEV, lower-end vertebra. *P < 0.05 vs. AV group; **P < 0.01 vs. AV group; ***P < 0.05 vs. AV ± 1 group.

## Discussion

HU value was considered to be a effective method to evaluate bone quality in many studies. Correlations of HU value with *T*-score have been reported, and it has been proposed as the primary criterion in the diagnosis of osteoporosis when the HU value at the L1 vertebral body was less than 110 (28, 29). Christensen et al*.* found that HU value at the proximal femur could be used to predict the risk of fracture, and a decline in HU value was closely related to the occurrence of fracture (30). HU value shows higher reliability and accuracy than traditional methods and can be used to evaluate the bone quality effectively and individually of every vertebra included in CT films (23–25).

In our study, we found that vertebral-body bone quality in the apical region of the structural curve in AIS patients was decreased when compared to the controls. Abnormal bone metabolism was considered to be an important factor in the pathogenesis of AIS (1, 9, 31). In a previous study, a significant difference in the bone mineral density between patients with AIS and non-affected paired controls was proven (32). Li et al. reported that AIS patients had poorer bone mineral density of the bilateral femoral neck than controls (13), and lower bone volume from the histological sections of the spinous process taken from AIS patients was found (33). Besides, Almomen et al. reported that female AIS patients with greater higher Cobb angles exhibited a significantly higher risk of low bone density (34). Our study is the first to use the HU value obtained from CT scans to evaluate the bone quality of vertebral bodies in AIS patients.

The asymmetric bony growth of vertebral bodies in AIS patients had been previously reported (19, 35–39). In our study, we evaluated bone quality using HU value and found the asymmetrical bone quality changes between the concave and convex sides of thoracic vertebral bodies in the AV-2, AV-1, AV, AV + 1, and AV + 2 regions of the structural curve in AIS patients. Besides, the bone quality of the convex side of vertebral bodies was significantly lower than that in the concave side. In addition, asymmetrical change in vertebral-body bone quality was most significant in the apical region. Although the mechanism was still unclear, it suggested that there was an asymmetrical change during the development of the skeleton system in AIS patients. In a previous study, the average width of pedicle was smaller in the non-structural curve than that in the structural curve in AIS patients (19). In our study, asymmetric change between the concave and convex sides was found in the region of structural curve but no significant difference in non-structural curve with a *p*-value larger than 0.05. The non-structural curve referred to the temporary and compensable curve without structural changes, which indicated that the change existed primarily in the structural curve. Moreover, it remains elusive whether a significant difference between concave and convex sides would be shown with a larger Cobb angle of the non-structural curve in AIS patients, and further studies are needed.

In surgery to correct AIS deformities, choosing the suitable screw could be important (40, 41). As known, the length and width of pedicle were generally considered to be the major factors in the choice of pedicle screw fixation during a deformity correction surgery (42, 43). Meanwhile, in previous studies, the thickness of cortical bone of pedicle had been reported to be an important factor for enhancing holding force of pedicle screw, and the screw stability depends on the structural characteristics of the pedicle (44–46). Besides, the quality of cancellous bone was also considered to be an influencing factor on the holding force of pedicle screws. Lower bone mass was considered as an affected factor of pedicle screw loosening, and regional HU value of the screw trajectory could be a strong predictor of long-term screw fixation (47, 48). Zou et al*.* reported that HU value measured on CT was an independent predictor for pedicle screw loosening, and lower HU value was significantly correlated with higher risk of screw loosening (49, 50). Another study showed that anti-osteoporosis treatment could achieve strong pedicle screw fixation effectively with an increase in bone mineral density around the screw assessed by QCT (51).Our results found lower bone quality in the convex vs. the concave side in the AV, AV ± 1, and AV ± 2 regions of the structural curve in AIS patients, suggesting that surgeons should exercise increased vigilance when selecting pedicle screw dimensions, a thicker and longer pedicle screw should be better to provide stronger holding force for internal fixation on the convex side during surgery when the width and length were suitable.

This study had the following limitations. Our results can not be applied to males because only female subjects were included in our study. Additionally, only Lenke 1A AIS patients were included. It would be ideal if we could repeat the same measurements in AIS patients of all other Lenke types. Furthermore, this is a single-center study and the entire study cohort was recruited from the southern region of China, which limits generalizability to other geographic locations, including the differences of temperature and elevation.

## Conclusions

Based on HU value obtained from CT scans of AIS patients, the bone quality of vertebral bodies in the apical region of the structural curve was significantly decreased compared with other regions, and asymmetrical changes were found between the concave and convex sides of vertebral bodies. Further, we found that the asymmetry was most significant in the apical region. In terms of application, thicker and longer pedicle screws should be chosen to provide stronger holding force for internal fixation on the convex side during surgery.

## Data Availability

The original contributions presented in the study are included in the article/**Supplementary Material**, further inquiries can be directed to the corresponding author/s.
