# Supplementary material for: Evaluating bone quality and asymmetrical aplasia of the thoracic vertebral body in Lenke 1A adolescent idiopathic scoliosis using hounsfield units
Source: Front Surg. 2022 Oct 31;9:1028873. doi: 10.3389/fsurg.2022.1028873 (PMC9659626; doi:10.3389/fsurg.2022.1028873)
Supplement: Supplementary file 1 [file Table2.docx]

**Supplementary Figures and Figure legends**


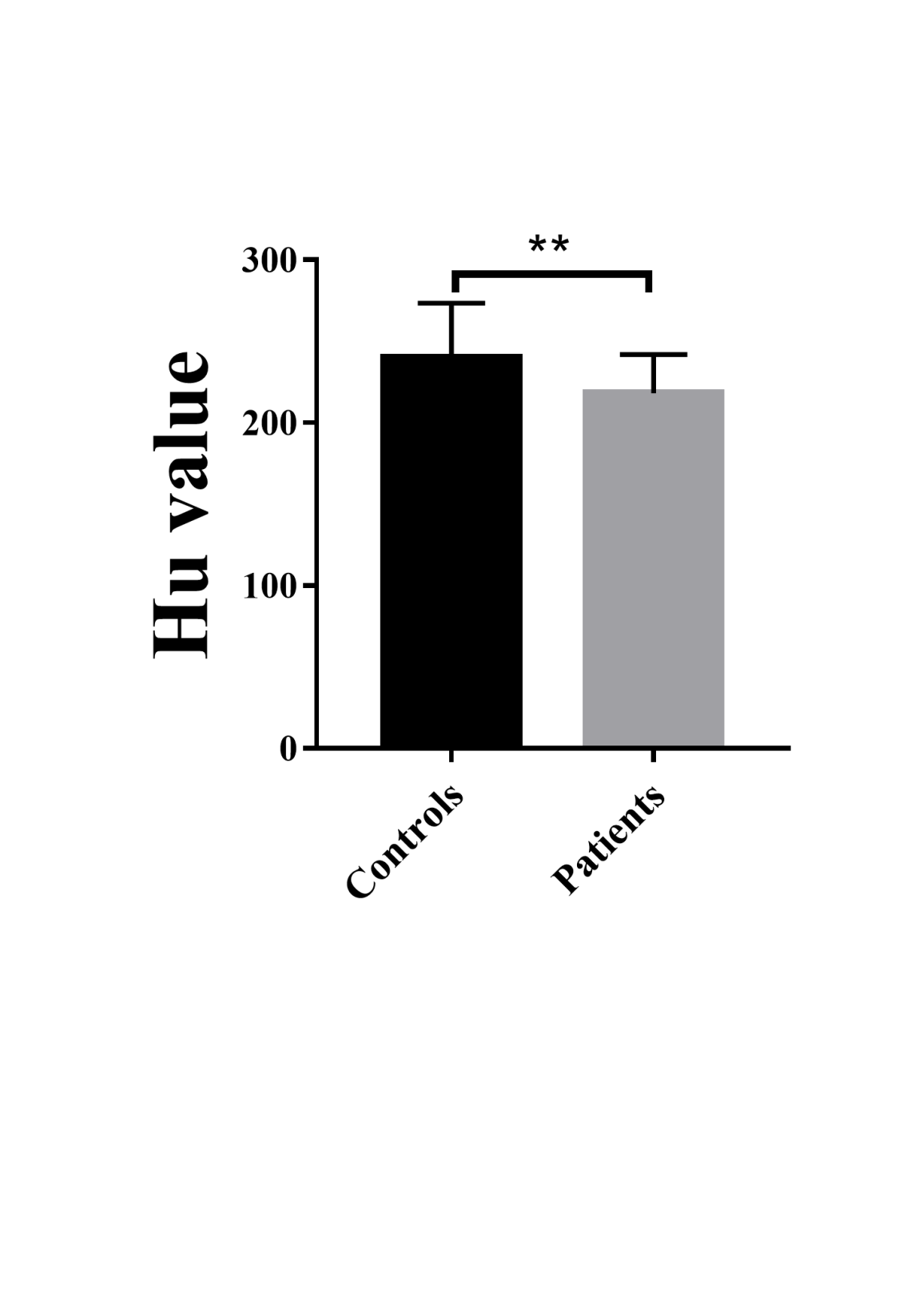


**Supplementary Figure 1.** Comparison of average HU value between the controls and the structural curves’ regions in Lenke 1A AIS patients. Total 30 patients with Lenke type 1A AIS and 30 paired controls were included in this study. ***P* < 0.01 *vs.* control group.


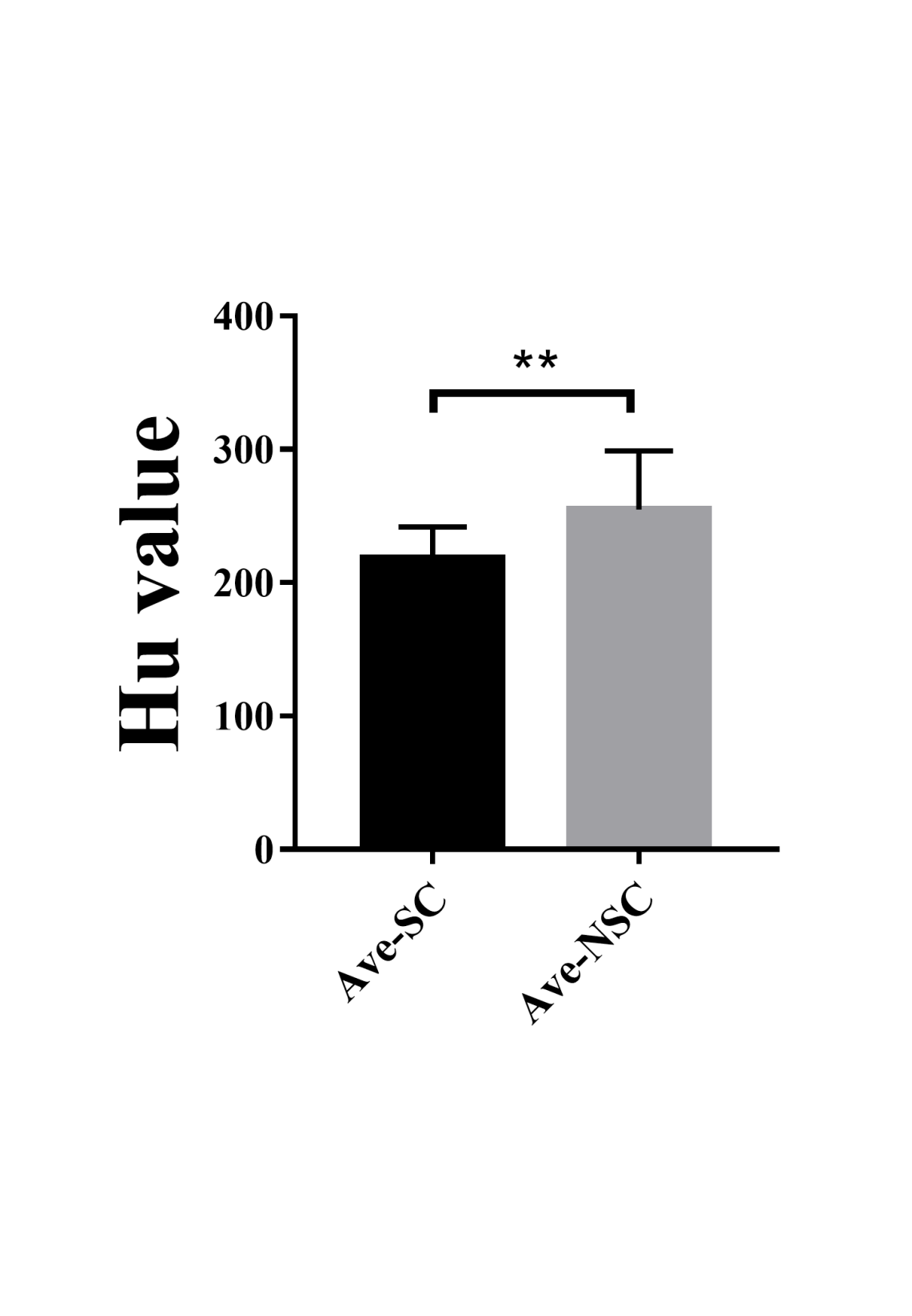


**Supplementary Figure 2.** Comparison of average HU value between the structural curves’ regions and the non-structural curves’ region in Lenke Type 1A AIS patients. Total 30 patients with Lenke type 1A AIS and 30 paired controls were included in this study. ***P* < 0.01 *vs.* control group.
